# Identification of fungal dihydrouracil-oxidase genes by expression in *Saccharomyces cerevisiae*

**DOI:** 10.1007/s10482-022-01779-9

**Published:** 2022-10-14

**Authors:** Jonna Bouwknegt, Aurin M. Vos, Raúl A. Ortiz Merino, Daphne C. van Cuylenburg, Marijke A. H. Luttik, Jack T. Pronk

**Affiliations:** Department of Biotechnology, Delft University of Technology, van der Maasweg 9, 2629 HZ Delft, The Netherlands

**Keywords:** Dihydroorotate dehydrogenase, Dihydropyrimidine dehydrogenase, Dihydropyrimidine oxidase, Counter-selectable marker genes, 5-fluorodihydrouracil, Dihydrothymine

## Abstract

Analysis of predicted fungal proteomes revealed a large family of sequences that showed similarity to the *Saccharomyces cerevisiae* Class-I dihydroorotate dehydrogenase Ura1, which supports synthesis of pyrimidines under aerobic and anaerobic conditions. However, expression of codon-optimised representatives of this gene family, from the ascomycete *Alternaria alternata* and the basidiomycete *Schizophyllum commune*, only supported growth of an *S. cerevisiae ura1*Δ mutant when synthetic media were supplemented with dihydrouracil. A hypothesis that these genes encode NAD(P)^+^-dependent dihydrouracil dehydrogenases (EC 1.3.1.1 or 1.3.1.2) was rejected based on absence of complementation in anaerobic cultures. Uracil- and thymine-dependent oxygen consumption and hydrogen-peroxide production by cell extracts of *S. cerevisiae* strains expressing the *A. alternata* and *S. commune* genes showed that, instead, they encode active dihydrouracil oxidases (DHO, EC1.3.3.7). DHO catalyses the reaction dihydrouracil + O_2_ → uracil + H_2_O_2_ and was only reported in the yeast *Rhodotorula glutinis* (Owaki in J Ferment Technol 64:205–210, 1986). No structural gene for DHO was previously identified. DHO-expressing strains were highly sensitive to 5-fluorodihydrouracil (5F-dhu) and plasmids bearing expression cassettes for DHO were readily lost during growth on 5F-dhu-containing media. These results show the potential applicability of fungal DHO genes as counter-selectable marker genes for genetic modification of *S. cerevisiae* and other organisms that lack a native DHO. Further research should explore the physiological significance of this enigmatic and apparently widespread fungal enzyme.

**Supplementary Information:**

The online version contains supplementary material available at 10.1007/s10482-022-01779-9.

## Introduction

**Fig. 1:**
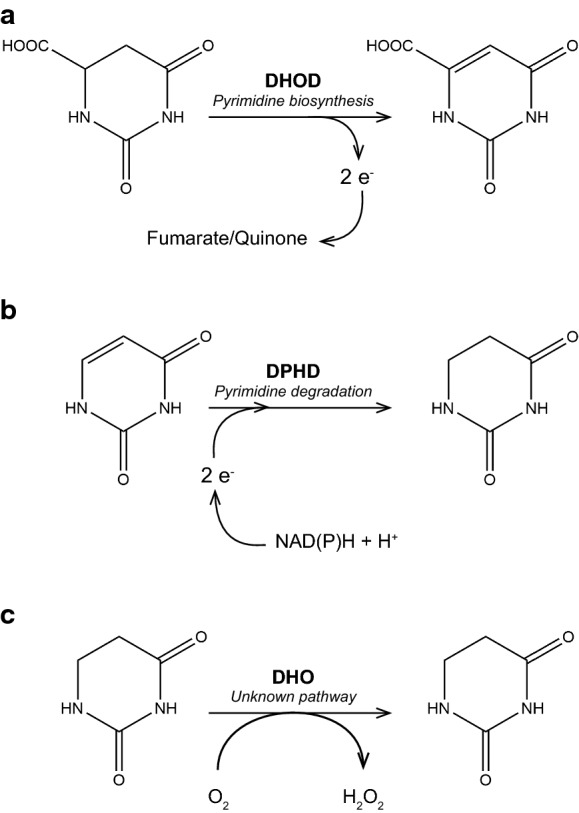
Reactions catalysed by dihydroorotate dehydrogenase, dihydropyrimidine dehydrogenase and dihydrouracil oxidase. **a**. In pyrimidine biosynthesis, fungal dihydroorotate dehydrogenase catalyses the oxidation of dihydroorotate orotate and donates electrons to the quinone pool of the respiratory chain (Class-II DHOD, but see (Bouwknegt et al. 2021)) or to fumarate (Class-I DHOD). **b.** In the reductive pathway for pyrimidine degradation, dihydropyrimidine dehydrogenases (DPHD) catalyse the reduction of uracil to dihydrouracil, using NAD(P)H + H^+^ as electron donor. **c.** Dihydrouracil oxidase (DHO), which has only been described in *Rhodotorula glutinis* (Davis et al. 1984; Owaki et al. 1986), catalyses oxidation of dihydrouracil with molecular oxygen and forms hydrogen peroxide

Cellular contents of the pyrimidines cytidine, thymine and uracil are the net result of uptake, de novo synthesis, salvage pathways and degradation (di Carlo et al. 1952; O’Donovan and Neuhard 1970). Whereas pyrimidine biosynthesis is highly conserved across all domains of life (O’Donovan and Neuhard 1970), pyrimidine degradation can occur via at least four different pathways (Hayaishi and Kornberg 1952; Vogels and van der Drift 1976; Loh et al. 2006; Andersen et al. 2008). The conserved pyrimidine biosynthesis pathway and the reductive pathway for pyrimidine degradation that occurs in most eukaryotes (Vogels and van der Drift 1976; Zrenner et al. 2006) involve similar, reversible redox reactions (Moffatt and Ashihara 2002). In pyrimidine biosynthesis, dihydroorotate dehydrogenase (DHOD) oxidises dihydroorotate to orotate (Fig. 1A), while the reductive pyrimidine degradation pathway starts with reduction of uracil to dihydrouracil (Fig. 1B) by NAD(P)^+^-dependent dihydropyrimidine dehydrogenases (DHPD), whose protein sequences show similarity to those of DHODs (Rowland et al. 1997; Dobritzsch et al. 2001). The DHPDs active in pyrimidine degradation, as well as the DHODs active in pyrimidine synthesis, are flavoproteins.

Genetic defects in human pyrimidine catabolism have been implicated in neurological disorders and mainly affect DHPD (Berger et al*.* 1984; van Kuilenburg et al. 1999). Furthermore, human DHPD plays a key role in resistance to the anticancer drug 5-fluorouracil (5-FU) by reducing it to the less toxic 5-fluorodihydroxyuracil (Heggie et al. 1987; Hull et al. 1988; Van Gennip et al. 1997; van Kuilenburg et al. 1999). Eukaryotic DHPDs, encoded by mammalian *DPYD* orthologs and plant *PYD1* orthologs, have been isolated and characterised (Smith and Yamada 1971; Podschun et al. 1989; Zrenner et al. 2009). They are homodimers containing one FAD, one FMN and four [4Fe-4S] clusters (Podschun et al. 1989; Lu et al. 1992). After reduction of the FAD cofactor with NADPH, electrons are transferred to FMN via the [4Fe-4S] clusters and FMNH_2_ reduces uracil or thymine, yielding dihydrouracil or dihydrothymine, respectively (Podschun et al. 1990).

Most fungi harbour homodimeric Class-II DHODs, which are targeted to the outside of the inner mitochondrial membrane (Rawls et al. 2000). These enzymes, which are orthologs of the Ura9 proteins in yeasts such as *Lachancea kluyveri* and *Ogataea parapolymorpha,* donate electrons to the quinone pool of the mitochondrial respiratory chain (Gojković et al. 2004; Fonseca et al. 2008; Riley et al. 2016). As a consequence, the large majority of fungi require oxygen for pyrimidine synthesis (Nagy et al. 1992). The facultative anaerobic *Saccharomyces* species and a small number of closely related Saccharomycotina yeasts, including facultatively anaerobic as well as oxygen-requiring species, instead harbour Class I-A DHODs (Ura1 in *S. cerevisiae*). These enzymes are soluble homodimers with one FMN domain per subunit and use fumarate as electron acceptor (Zameitat et al. 2007).

Presence of Ura1 orthologs, which in Saccharomycotina are proposed to have been acquired by horizontal gene transfer (HGT) from lactic acid bacteria (Gojković et al. 2004), were long considered to be essential for anaerobic pyrimidine prototrophy of fungi (Hall et al. 2005). This notion was first questioned when the facultatively anaerobic yeast *Dekkera bruxellensis* was shown to only contain a DHOD gene with sequence similarity to yeast Class-II DHOD genes (Woolfit et al. 2007; Piškur et al. 2012). We recently showed that expression of Class-II DHOD genes from *D. bruxellensis*, from obligately anaerobic Neocallimastigomycota, or from the facultatively anaerobic fission yeast *Schizosaccharomyces japonicus*, supported anaerobic growth of *S. cerevisiae ura1*Δ strains without pyrimidine supplementation (Bouwknegt et al. 2021). These results indicated that acquisition of a Class-I DHOD by HGT is not the only mechanism by which fungi can evolve for anaerobic pyrimidine prototrophy.

While searching fungal proteomes for homologs of *S. cerevisiae* Ura1 that might indicate previously unidentified HGT events leading to the acquisition of Class-I DHODs, we encountered an unexpectedly large number of putative proteins in ascomycetes and basidiomycetes that showed similarity to Class-I DHOD sequences. A widespread occurrence of Ura1 orthologs in fungi was unexpected in view of the previously reported connection of Class-I DHOD genes to anaerobic growth and the proposed role of HGT in their acquisition. To investigate the biochemical function of the proteins encoded by this previously unexplored fungal gene family, we expressed representatives from the ascomycete *Alternaria alternata* and the basidiomycete *Schizophyllum commune* in a *ura1Δ* strain of *Saccharomyces cerevisiae*. Since *S. cerevisiae* and other post-whole-genome-duplication Saccharomycotina yeast species lost the ability to degrade pyrimidines (Andersen et al. 2006), expression in *ura1Δ* strains enabled analysis of DHOD and DHPD activity of the encoded proteins as well as an assessment on the requirement for oxygen of their activities. Growth experiments and enzyme activity assays in cell extracts showed that the *A. alternata* and *Sch. commune* genes encoded dihydrouracil oxidases (DHO) that catalyse oxygen-dependent oxidation of dihydrouracil to uracil (Fig. 1C) and also showed activity with dihydrothymine. Based on the reported ability of human DHPD to convert 5-fluorodihydrouracil to the toxic compound 5-fluorouracil (Heggie et al. 1987), we tested whether fungal *dho* genes can be applied as counter-selectable marker genes for genetic modification of *S. cerevisiae*.

## Results

### Genome analysis reveals a large cluster of uncharacterised fungal protein sequences with similarity to yeast Ura1

**Fig. 2:**
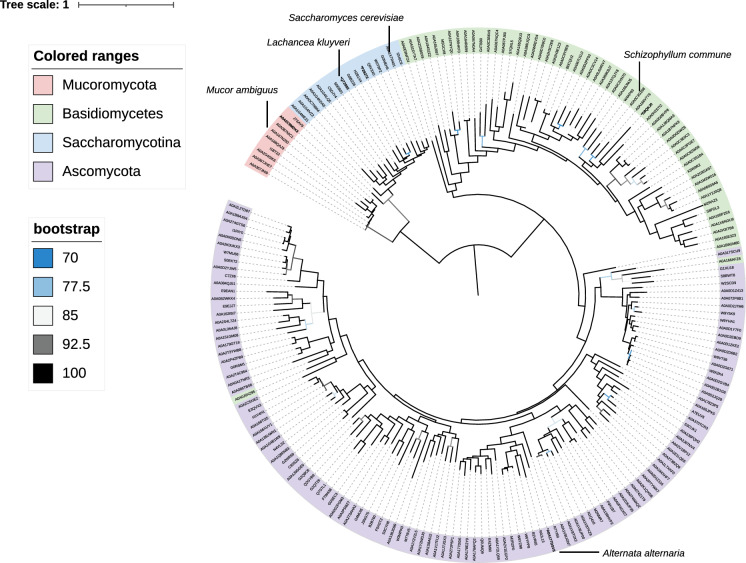
Maximum likelihood phylogenetic tree of *L. kluyveri* (LkUra1; dihydroorotate dehydrogenase) fungal orthologs. A search for orthologs of Class I-A dihydroorotate dehydrogenases using *Lachancea kluyveri* LkUra1 (UniProt KB accession number: Q7Z892) as query resulted in 203 proteins (Dataset S01). Sequence identifiers corresponding to the phyla Basidiomycota, Ascomycota and Mucoromycota are indicated in color. Sequences that were functionally analysed in this study, as well as the the characterised Ura1 proteins of *L. kluyveri* (Gojković et al. 2004), and *S. cerevisiae* (Zameitat et al. 2007) are indicated with the corresponding species name. The tree was midpoint rooted, and the raw phylogenetic tree is provided in Dataset S02. Bootstrap values and species can be accessed from: https://itol.embl.de/tree/8384480104951618225493

To search fungal proteomes for possible Class-I-A DHODs resembling the Ura1 proteins of *S. cerevisiae* and closely related Saccharomycotina yeasts, the amino-acid sequence of the Class-I-A DHOD LkUra1 from *Lachanchea kluyveri* was used as query for a HMMER search against fungal proteomes available from Uniprot (The Uniprot Consortium 2019). This search yielded 203 putative Ura1 orthologs (Fig. 2, Dataset S01, S02). Eight of these proteins originating from Mucoromycotina showed a strong homology to the well-known Class-I-A DHODs from *S. cerevisiae, L. kluyveri* and other Saccharomycotina yeasts (Fig. 2), despite the phylogenetic distance of these groups (Li et al. 2021). This observation coincided with the reported ability of dimorphic *Mucor* species to grow anaerobically without pyrimidine supplementation (Bartnicki-Garcia and Nickerson 1962; Jeennor et al. 2006). A large group of additional putative orthologs of LkUra1 in Ascomycota and Basidiomycota (Fig. 2) was unexpected, since the only known fungal Class-I-A DHODs are proposed to have been obtained by horizontal gene transfer (Gojković et al. 2004). Most of these sequences were found in proteomes of aerobic fungi that also contain a Class-II DHOD (Bouwknegt et al. 2021). This observation raised the question whether and why these organisms harbour different, seemingly redundant DHODs. The group of putative Ura1-orthologs showed two large clusters, which almost exclusively consisted of sequences from either ascomycetes or basidiomycetes. Only two sequences from basidiomycetes (*Exidia glandulosa;* A0A166AFZ8 and *Cutaneotrichonsporon oleaginosum;* A0A0J0XZ95) clustered with those from ascomycetes (Fig. 2). We therefore hypothesised that, rather than DHODs, this group of LkUra1-like protein sequences in ascomycetes and basidiomycetes might comprise proteins with a related function, such as DHPDs (Dobritzsch et al. 2001).

### Putative Ura1-orthologs from *A. alternata* and *Sch. commune* are not DHODs

To investigate the biochemical activity of the group of fungal proteins with sequence similarity to *S. cerevisiae* Ura1, a representative from an ascomycete (*Alternaria alternata*; Aa; A0A177DSV5) and a basidiomycete (*Schizophyllum commune*; Sc; D8QKJ0) were selected (Fig. 2). An alignment of these sequences with those of Ura1 orthologs from *L. kluyveri* and *S. cerevisiae* confirmed the strong similarity inferred from the phylogenetic analyses (SI Figure S1). Conserved amino-acid residues involved in flavin cofactor binding, as well as an active-site cysteine residue that is strongly conserved in Class-I-A DHODs occurred in all four sequences. Codon-optimised coding regions of the corresponding genes, further referred to as Aa*dho* and Sc*dho* (see below), respectively, were expressed in the uracil-auxotrophic *S. cerevisiae* strain IMK824 (*ura1*∆), which lacks DHOD activity and whose growth on synthetic medium with glucose (SMD) should therefore require supplementation with uracil (SMD + ura; Fig. 1; Hall et al. 2005). Since the *S. cerevisiae* genome does not encode a DHPD (Andersen et al. 2006), in vivo DHOD activity of the heterologous proteins should complement the *ura1*∆ mutation on SMD, while in vivo DHPD activity should only do so upon supplementation of media with dihydrouracil (SMD + dhu; Fig. 1). As anticipated, the reference *S. cerevisiae* strain IMX585 (*URA1*) showed the same specific growth rate (0.37 h^−1^) on all three media, while strain IMK824 (*ura1*∆) only grew on SMD + ura supplemented with uracil (Table 1). The latter, IMK824, grew slightly slower on SMD + ura than the reference strain (Table 1), probably reflecting a limitation in uracil import (Pronk 2002). Strains IMI433 (*ura1*∆*::*Aa*dho*) and IMI434 (*ura1*∆::Sc*dho*) did not show growth on SMD (Table 1), indicating that the heterologous fungal genes were either not functionally expressed in *S. cerevisiae* or did not encode functional DHODs. The subsequent observation that both strains showed a specific growth rate of 0.34 h^−1^ on SMD + dhu, which did not support growth of the *ura1*∆ strain IMK824 (Table 1), led us to hypothesise that Aa*dho* and Sc*dho* both encode fungal DHPDs.

**Table 1:** Complementation by uracil (ura) or dihydrouracil (dhu) of uracil-auxotrophic *S. cerevisiae* strains expressing fungal proteins with homology to Ura1

Strain	Relevant genotype	SMD	SMD + ura	SMD + dhu
IMX585	*URA1*	0.37 ± 0.00	0.37 ± 0.00	0.37 ± 0.01
IMK824	*ura1*Δ	n.a	0.35 ± 0.00	n.a
IMI433	*ura1*Δ::Aa*dho*	n.a	0.37 ± 0.00	0.34 ± 0.00
IMI434	*ura1*Δ::Sc*dho*	n.a	0.35 ± 0.00	0.34 ± 0.01

Specific growth rates were measured in duplicate shake-flask cultures for each combination of strain and medium composition, data are represented as average ± mean deviation. SMD, synthetic medium with glucose; SMD + ura, SMD supplemented with 1.5 g L^−1^ uracil; SMD + dhu, SMD supplemented with 2.5 g L^−1^ dihydrouracil. n.a. (not applicable) indicates that no exponential growth was observed

### *S. cerevisiae* strains expressing Aa*dho* or Sc*dho* do not oxidise dihydrouracil in anaerobic cultures

**Fig. 3:**
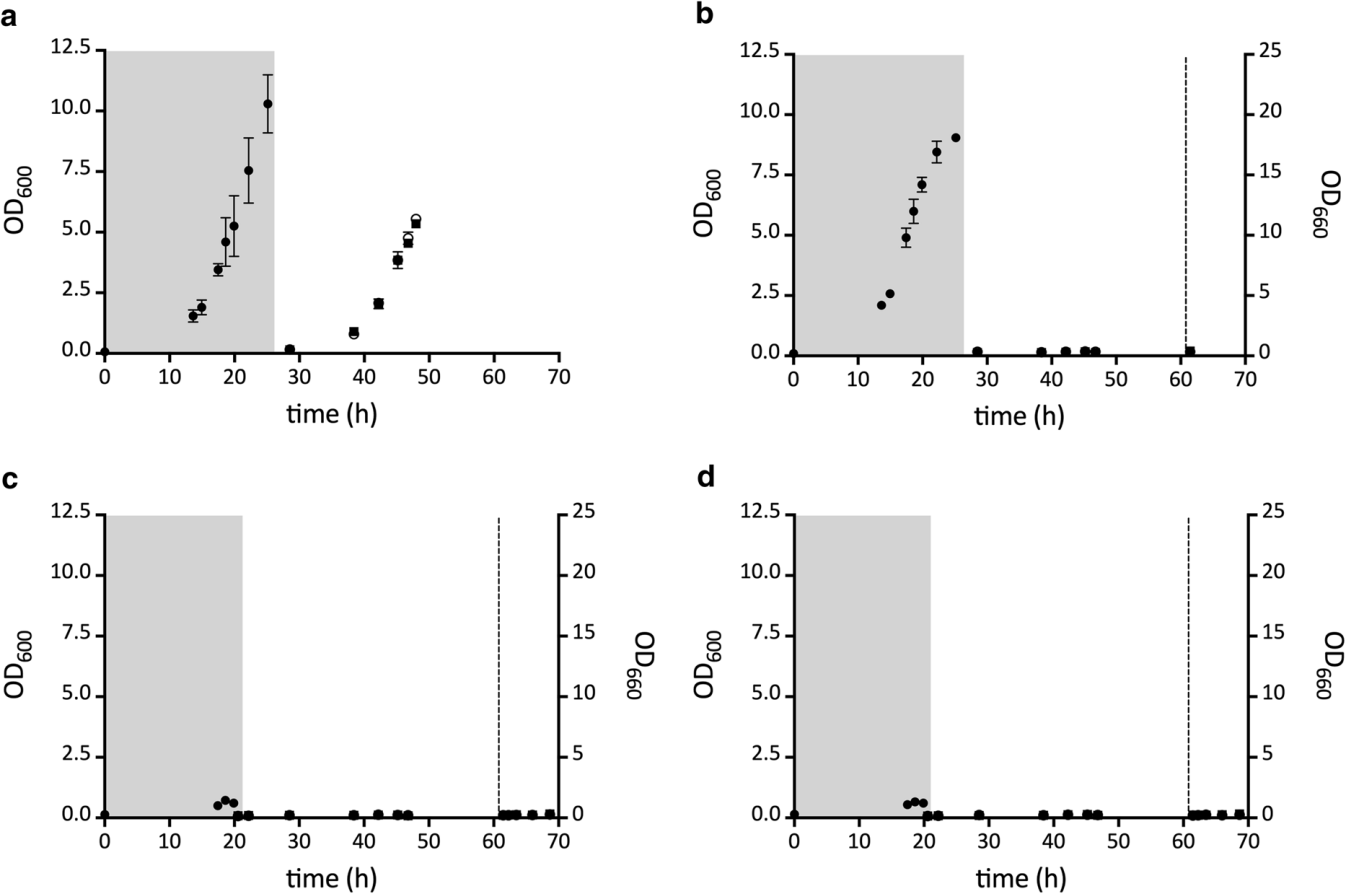
Anaerobic growth curves of *S. cerevisiae* strains expressing genes encoding putative Ura1 orthologs from *Alternaria alternata* (Aa*dho*) and *Schizophyllum commune* (Sc*dho*). Strains were pre-grown anaerobically (Black circle) on medium with a limiting amount of uracil (strain IMK824 (*ura1Δ*)) or dihydrouracil (strains IMX585 (*URA1*), IMI433 (*ura1Δ::*Aa*dho*) and IMI434 (*ura1Δ::*Sc*dho*) to deplete intracellular pyrimidine reserves (grey boxes; Mooiman et al. 2021). Strains were then transferred to fresh media with dihydrouracil (SMD + dhu; Black Square) or without dihydrouracil (SMD; White circle). After 62 h (dotted line), strains IMI433 and IMI434 were transferred to aerobic conditions. **a**; *S. cerevisiae* IMX585 (*URA1*), **b**; strain IMK824 (*ura1Δ*), **c;** strain IMI433 (*ura1Δ*::Aa*dho*), **d**; strain IMI434 (*ura1Δ*::Sc*dho*). Data represent the average and mean deviation of duplicate cultures

To test whether dihydrouracil oxidation by strains IMI433 (*ura1*∆*::*Aa*dho*) and IMI434 (*ura1*∆::Sc*dho*) required oxygen, their anaerobic growth was compared with that of the reference strains IMX585 and IMK824 (*ura1Δ*). After aerobic pre-cultivation on permissive synthetic media, washed cell suspensions were transferred to second-stage anaerobic pre-cultures on SMUD with a limiting amount of uracil (SMUD + ura0.1) or dihydrouracil (SMUD + dhu0.1). Upon a subsequent transfer, strain IMK824 (*ura1*∆) did not grow on SMUD unless uracil was supplemented (Fig. 3). This result confirmed that the pre-cultivation procedure had successfully depleted any intracellular reserves of pyrimidines. When the *S. cerevisiae* strains expressing either Aa*dho* or Sc*dho* were similarly transferred to an anaerobic culture, no growth on SMUD or SMUD + dhu was observed (Fig. 3). However, when anaerobic cultures of these strains on SMD + dhu were subsequently transferred to aerobic conditions, instantaneous growth occurred (Fig. 3). These results showed that oxygen is required for in vivo oxidation of dihydrouracil by Aa*dho* and Sc*dho*-encoded proteins.

### Aa*dho* and Sc*dho* encode dihydrouracil oxidases

**Fig. 4:**
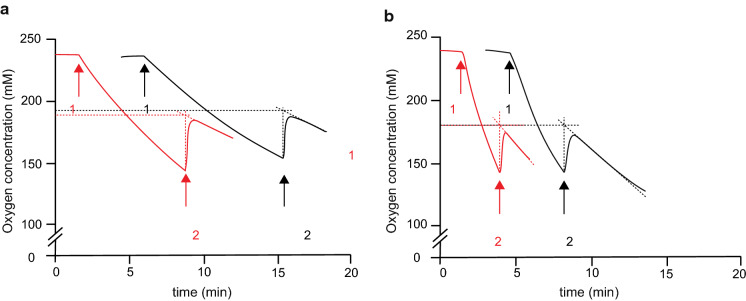
Substrate-dependent oxygen consumption and catalase-dependent oxygen formation by cell extracts of *S. cerevisiae* strains expressing fungal dihydrouracil oxidases*.* Reaction volumes of 4 mL contained cell extracts of **a.** *S. cerevisiae* IME664 (Aa*dho*), **b.** *S. cerevisiae* IME665 (Sc*dho*). Oxygen concentration was monitored with a Clark-type electrode. Arrows indicate the following additions: **1**; 1 mM dihydrothymine (black lines) or 1 mM dihydrouracil (red lines), **2**; approximately 500 U catalase

The observed oxygen requirement for dihydrouracil oxidation of *S. cerevisiae* strains expressing Aa*dho* or Sc*dho* raised the possibility that these genes might encode dihydrouracil oxidases (DHO; Fig. 1, Davis et al. 1984). To test this hypothesis, expression cassettes were introduced in an *S. cerevisiae ura1*∆ background on multicopy (mc) plasmids. Cell extracts of the resulting strains IME664 (*ura1*∆ mcAa*dho*) and IME665 (*ura1*∆ mcSc*dho*) were then used for enzyme activity assays*.* Extracts of strains IME664 and IME665 showed dihydrouracil-dependent oxygen consumption (Table 2; 29.6 ± 6.5 and 246 ± 13 nmol O_2_ (mg protein)^−1^ min^−1^_,_ respectively) as well as dihydrothymine-dependent oxygen consumption (46.1 ± 8.6 and 340 ± 22 nmol O_2_ (mg protein)^−1^ min^−1^). No significant rates of oxygen consumption were observed upon addition of dihydrouracil or dihydrothymine to cell extracts of the reference strains CEN.PK113-7D (*URA1*) or IMK824 (*ura1*∆) (Table 2). When catalase, which catalyses the reaction 2 H_2_O_2_ → O_2_ + 2 H_2_O, was added during oxidation of dihydrouracil by cell extracts of strains IME664 or IME665, the oxygen concentration in the reaction mixture immediately increased (Fig. 4). This increase corresponded to about 40% of the initially consumed oxygen rather than the theoretically predicted 50%. This observation is likely to be due to the presence of some catalase in the yeast cell extracts. These results supported the hypothesis that Aa*dho* and Sc*dho* are structural genes encoding active fungal DHO enzymes.

**Table 2:** Substrate-dependent oxygen-uptake activities of cell extracts of *S. cerevisiae* strains expressing Aa*dho* or Sc*dho*

Strain	Relevant genotype	Activity (nmol O_2_·mg protein^−1^·min^−1^)	
		dihydrouracil	dihydrothymine
CEN.PK113-7D	*URA1*	< 2	< 2
IMK824	*ura1*∆	< 2	< 2
IME664	*ura1*∆ mcAa*dho*	29.6 ± 6.5	46.1 ± 8.6
IME665	*ura1*∆ mcSc*dho*	246 ± 13	340 ± 22

Activities were determined in cell extracts by monitoring oxygen upon addition of 1 mM dihydrouracil or 1 mM dihydrothymine. Experiments were performed with a Clark-type oxygen electrode, in a 4 mL reaction chamber kept at 30 °C and containing 0.1 M potassium phosphate buffer (pH 7.5) with 2 mM MgCl_2_. Activities are presented as average and mean deviation of measurements on two independently prepared cell extracts for each strain

DHO activity was previously only reported in two studies on *Rhodotorula glutinis* (Davis et al. 1984; Owaki et al. 1986). Since the proteome of *R. glutinis* is not available from Uniprot, no Ura1-ortholog of this yeast was included in our phylogenetic analysis. However, homologous proteins were found in *Rhodotorula* species, *R. taiwanensis* (A0A2S5B0X9) and *R. graminis* (A0A194S2Z2) as well as in a predicted protein sequence derived from the *R. glutinis* genome sequence available at JGI Mycocosm (Grigoriev et al. 2014). Although, similar to our results (Table 2), the early studies on *R. glutinis* indicated activity with dihydrouracil as well as dihydrothymine, the enzyme activity was classified as a dihydrouracil oxidase (EC1.3.3.7). We therefore propose the name *dho* (for dihydrouracil oxidase) for the genes encoding A0A177DSV5 from *Alternaria alternata* (Aa*dho*) and D8QKJ0 from *Schizophyllum commune* (Sc*dho*).

### Aadho and Scdho can be used as dominant counter-selectable marker genes in uracil-auxotrophic *S. cerevisiae* mutants

**Fig. 5:**
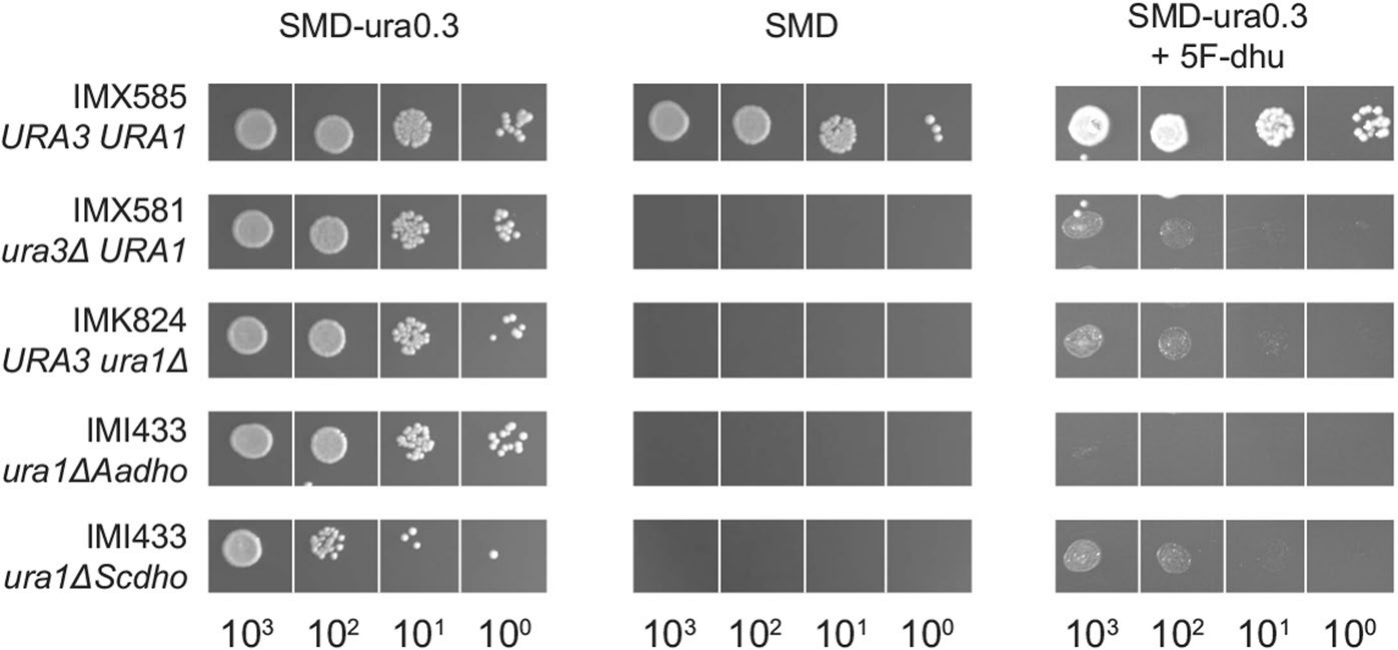
Effect of 5-fluorodihydrouracil on growth of *S. cerevisiae* strains expressing fungal dihydrouracil oxidases. Strains IMX585 (*URA1*), IMK824 (*ura1* null mutant) and IMX581 (*ura3* null mutant), IMI433 (*ura1Δ*::Aa*dho*) and IMI434 (*ura1Δ*::Sc*dho*) were plated on synthetic medium with 20 g L^−1^ glucose (SMD; left), medium supplied with 150 mg L^−1^ uracil (SMD + ura; middle) and SMD supplied with 50 mg L^−1^ dihydrouracil and 2.5 g L^−1^ of 5-fluorodihydrouracil (SMD + ura0.3 + 5F-dhu; right) and incubated aerobically at 30 °C for 2 days. Duplicate experiments yielded the same results

Based on the observation that expression of the two fungal *dho* genes enabled uracil-auxotrophic mutants of *S. cerevisiae* to grow on SMD + dhu, we explored whether they might be applicable as counter-selectable dominant marker genes in such strains. To this end, we investigated whether the DHOs encoded by Aa*dho* and Sc*dho* convert 5-fluorodihydrouracil (5F-dhu) into the toxic compound 5-fluorouracil (5-FU), as has been described for mammalian DHPDs (Shiotani and Weber 1981; Heggie et al. 1987). The two DHO-expressing strains *S. cerevisiae* IMI433 and IMI434, the uracil-auxotrophic strains IMX581 (*ura3*∆) and IMK824 (*ura1*∆) and the reference strain IMX585 (*URA1*) were plated on SMD + ura and on SMD with 5F-dhu and uracil. Growth of the reference strain IMX585 was not substantially affected by 5F-dhu (Fig. 5). In contrast, 5F-dhu strongly inhibited growth of the DHO-expressing strains and even completely blocked growth of strain IMI433 (*ura1*∆::Aa*dho*). Growth of the *ura1* and *ura3* null mutants IMK824 and IMX581 on SMD + ura was also inhibited by 5F-dhu, to similar extent as the inhibition observed for strain IMI434 (*ura1*∆::Sc*dho*). This observation suggests that 5F-dhu inhibits uracil uptake via the *S. cerevisiae* Fur4 transporter (Jund et al. 1988). The much stronger growth inhibition by 5F-dhu observed for strain IMI433 indicates that AaDho has activity towards this substrate while, based on the growth assays on plates, no conclusion could be drawn on whether the same holds for ScDho.

To explore counter selection, Aa*dho* and Sc*dho* were expressed in a *ura3* null mutant of *S. cerevisiae* from a plasmid that also carried a *URA3* marker gene. These strains were then tested for plasmid loss and viability after growth on SMD + ura with and without 5F-dhu. The reference strain IME426, which contained the *URA3*-carrying empty plasmid, showed the same viability after growth on SMD + ura with and without 5F-dhu (Table 3). The fraction of viable cells of this strain that had lost the plasmid after growth on SMD + ura with 5F-dhu was lower than after growth on SMD + ura (50% vs. 67%; Table 3). This observation was consistent with the hypothesis that 5F-dhu inhibits uracil uptake.

**Table 3:** Effect of 5-fluorodihydrouracil on viability and plasmid loss of *S. cerevisiae* strains expressing the fungal dihydrouracil oxidase genes Aa*dho* and Sc*dho*

Strain	Relevant genotype	CFU (%)		Plasmid loss (%)	
		SMD + ura	SMD + ura0.1 + 5F-dhu	SMD + ura	SMD + ura0.1 + 5F-dhu
IME426	*ura3-52* pUD63 (*URA3*)	84 ± 2	87 ± 1	67 ± 2	50 ± 1
IME410	*ura3-52* pUDE736 (*URA3* Aa*dho*)	92 ± 2	44 ± 6	61 ± 3	99 ± 0
IME414	*ura3-52* pUDE737 (*URA3* Sc*dho*)	91 ± 0	57 ± 5	60 ± 2	94 ± 1

Yeast strains were grown until late-exponential phase on either SMD with 1.5 g L^−1^ uracil (SMD + ura) or on SMD supplemented with both 0.15 g L^−1^ uracil (SMD + ura0.1) and 2.5 g L^−1^ 5F-dhu. Viability was calculated as the percentage of 768 cells plated on SMD + ura that formed colonies. Plasmid loss was calculated from the ratio of cell counts after plating on SMD and SMD + ura. Results are presented as the average ± mean deviation of data obtained with duplicate cultures for each strain

In contrast to the empty-plasmid control strain, *S. cerevisiae* strains harbouring plasmid-borne expression cassettes for Aa*dho* (IME410) or Sc*dho* (IME414) showed a 2.1-fold and 1.6-fold lower fraction of viable cells, respectively, after growth on SMD + ura with 5F-dhu than after growth on SMD + ura. This indication that 5F-dhu was toxic for both strains was even more strongly supported by the observation that, after growth on SMD + ura with 5F-dhu, strains IME414 (expressing Aa*dho*), IME410 (expressing Sc*dho*) showed plasmid losses of 99.4% and 93%, respectively. This difference was consistent with results from growth experiments with strains expressing a single copy of the *dho* genes, in which strain IMI433 showed a stronger growth inhibition by 5F-dhu than strain IME434 (Fig. 5). In contrast, DHO activities were higher in cell extracts of *S. cerevisiae* IME665, which expressed ScDho, than in the AaDho-expressing strain IME664 (Table 2). These results indicate that Aa*dho* can be effectively selected against by growth of *S. cerevisiae* strains in non-selective medium supplemented with 5F-dhu.

## Discussion

The anaerobic pyrimidine prototrophy of *Saccharomyces cerevisiae* has been attributed to the acquisition by horizontal gene transfer (HGT) of a Class I-DHOD-encoding gene from a lactic acid bacterium (Gojković et al. 2004). This study was originally initiated to explore whether similar HGT events involving Class I-DHOD-encoding gene occurred in fungi outside the Saccharomycotina subdivision. Consistent with a brief literature mention that *Mucor* DHOD resembles Class I-A DHODs (Oliver et al. 2016), we identified orthologs of Saccharomycotina Ura1 DHOD proteins in proteomes of Mucoromycetes. Strong sequence similarity of these proteins to Class-I DHODs from gram-negative Neisseriacaea (Table S1) supported occurrence of a second, independent bacterium-to-fungus transfer of a Class-I DHOD gene. Involvement in the reported pyrimidine-prototrophic anaerobic growth of *Mucor* species (Bartnicki-Garcia and Nickerson 1962) can be investigated by complementation studies with an *S. cerevisiae ura1*∆ strain.

Our search for potential HGT events involving Class-I DHODs unexpectedly identified a large cluster of ascomycete and basidiomycete sequences that all showed sequence homology with Class-I DHOD proteins. Expression of two genes encoding representatives from this cluster, one from an ascomycete and one from a basidiomycete, in an *S. cerevisiae ura1Δ* strain failed to complement its uracil prototrophy. However, the resulting strains did show oxygen-dependent growth when dihydrouracil was added to growth media (Table 1, Fig. 3). Despite their sequence similarity with Class-I DHOD from the yeasts *L. kluyveri* and *S. cerevisiae* (SI Figure S1), these fungal genes were found to encode dihydrouracil oxidases (DHO, EC1.3.3.7). Before this study, DHO activity had only been reported in two publications on the yeast *Rhodotorula glutinis* (Davis et al. 1984; Owaki et al. 1986) and no structural gene for DHO had been identified.

Our results show that fungal *dho* genes can be used as counter-selectable marker genes in uracil-auxotrophic *S. cerevisiae* strains. While use of 5-fluoroorotic acid already allows for efficient counterselection of the frequently used *URA3* marker gene in this yeast (Längle-rouault and Jacobs 1995), use of *dho* as marker gene should enable two consecutive selective transformations based on the *ura3* mutation. In a first transformation, *dho* gene can be used by selecting for dihydrouracil-dependent growth, after which *URA3* can still be used by selecting for growth in the absence of dihydrouracil. Simultaneous counterselection against *dho* and *URA3* could then potentially be performed in medium containing 5-fluorodihydrouracil and 5-fluoroorotic acid.

A phylogenetic analysis (Fig. 2) indicated that *dho* genes probably occur in a wide range of ascomycetes and basidiomycetes, which raises intriguing questions about the physiological role of DHO in fungi. Dihydrouracil and dihydrothymine, the substrates of DHO, are intermediates of the reductive pathway for pyrimidine degradation, which also provides β-alanine for pantothenate synthesis (Cambell 1956; Vogels and van der Drift 1976). In addition, ultraviolet radiation induces conversion of pyrimidines in DNA into dihydrouracil, dihydrothymine and dihydrocytosine (Wierzchowski and Schugar 1957; Yakovlev et al. 1995). Subsequent base- or nucleotide excision repair can lead to direct or indirect release of these dihydropyrimidines in cells (Venkhataraman et al. 2001). Studies on cancer cells and on in vitro DNA replication in *Xenopus* egg extracts showed that dihydropyrimidines cause DNA–protein crosslinking, interfere with DNA replication and cause transcriptional stress (Basbous et al. 2020). In mammalian cells, the zinc metalloprotein dihydropyrimidinase plays a key role in dihydropyrimidine detoxification (Brooks et al. 1979; Kikugawa et al. 1994). Dihydropyrimidinase in the yeast *L. kluyveri* was also shown to be a zinc metalloprotein (Dobritzsch et al. 2005) and, based on sequence similarity, this enzyme probably occurs in many other fungi (Dataset_S03). DHO might contribute to dihydropyrimidine detoxification when zinc limitation or other factors prevents optimal activity of dihydropyrimidinase.

Sequence similarity of fungal DHOs and Class-I-A DHODs includes shared conserved residues for flavin binding and in their putative catalytic sites (Figure S1), while no indications were found for binding of the four [4Fe-4S] clusters that occur in homodimeric mammalian DHDPs. In view of this sequence similarity, the reactions catalysed by Class-I-A DHOD and DHO (a flavin-mediated reduction of fumarate and an oxidase reaction, respectively) appear remarkably different. However, Class-I DHODs as well as quinone-dependent Class-II DHODs show low rates of oxygen-dependent dihydroorotate oxidation (Björnberg et al. 1997; Zameitat et al. 2007; Arakaki et al. 2008; Hey-Mogensen et al. 2014). Structure–function analysis should resolve the structural factors that determine their different electron donor and electron acceptor specificities of Class-I DHODs and DHOs and contribute to dissection of the evolutionary histories of these intriguing enzymes.

## Methods

### Growth media

Sterile synthetic medium with vitamins supplemented with 20 g L^−1^ D-glucose (SMD) for growth of *S. cerevisiae* was prepared as described previously (Verduyn et al. 1992). Anaerobic cultures were grown on synthetic medium with vitamins and urea as nitrogen source (SMUD), supplemented with the anaerobic growth factors Tween 80 and ergosterol (Luttik et al. 2000). Uracil was added to media as an autoclaved (121 °C, 20 min) 3.75 g L^−1^ solution, to final concentrations of either 0.15 g L^−1^ (SM(U)D + ura), 50 mg L^−1^ (SM(U)D + ura0.3) or 15 mg L^−1^ (SM(U)D + ura0.1). When indicated, dihydrouracil (Sigma-Aldrich, St. Louis MO) was added to media prior to sterilisation, to a concentration of 0.15 g L^−1^. Similarly, a filter-sterilised stock solution of 5-fluorodihydrouracil (5F-dhu; abcr GmbH, Karlsruhe, Germany) was added to sterile medium to a concentration of 2.5 g L^−1^ where indicated. Yeast extract-peptone-dextrose medium (YPD) was prepared as described previously (Mans et al. 2017). For selection of strains harbouring the *kanMX* gene, 200 mg L^−1^ geneticin (G418) was added to YPD. *Escherichia coli* cultures were grown on Lysogeny Broth (LB; Bertani 1951), autoclaved at 121 °C for 20 min and supplemented with 100 mg L^−1^ ampicillin (LB-amp). Solid media were prepared by adding 20 g L^−1^ Bacto Agar (BD Biosciences).

### Strains and maintenance

*S. cerevisiae* strains used in this study (Table 4) were derived from the CEN.PK lineage (Entian and Kötter 2007). For preparation of frozen stock cultures, yeast strains were pre-grown on SMD + ura (Verduyn et al. 1992). Plasmid-containing *E. coli* XLI-blue (Agilent Technologies, Santa Clara, CA, USA) strains were pre-grown at 37 °C and *S. cerevisiae* strains at 30 °C and at 200 rpm in an Innova incubator shaker (New Brunswick Scientific, Edison NJ). Stationary-phase cultures were supplemented with 30% (w/v) glycerol and stored at − 80 °C.

**Table 4:** *S. cerevisiae* strains used in this study

Strain	Relevant genotype	Reference
CEN.PK 113-5D	*MATa HIS3 LEU2 TRP1 ura3-52*	(Entian and Kötter 2007)
CEN.PK 113-7D	*MATa HIS3 LEU2 TRP1 URA3*	(Entian and Kötter 2007)
IMX581	*MATa HIS3 LEU2 TRP1 ura3-52 can1*∆*::cas9-natNT2*	Mans et al. (2015)
IMX585	*MATa HIS3 LEU2 TRP1 URA3 can1*∆*::cas9-natNT2*	Mans et al. (2015)
IMK824	*MATa HIS3 LEU2 TRP1 URA3 can1*∆*::cas9-natNT2 ura1*∆	This study
IMI433	*MATa HIS3 LEU2 TRP1 URA3 can1*∆*::cas9-natNT2 ura1*∆*::*Aa*dho*	This study
IMI434	*MATa HIS3 LEU2 TRP1 URA3 can1*∆*::cas9-natNT2 ura1*∆*::*Sc*dho*	This study
IME410	*MATa HIS3 LEU2 TRP1 ura3-52* pUDE737	This study
IME414	*MATa HIS3 LEU2 TRP1 ura3-52* pUDE736	This study
IME426	*MATa HIS3 LEU2 TRP1 ura3-52* pUD63	This study
IME664	*MATa HIS3 LEU2 TRP1 URA3 can1*∆*::cas9-natNT2 ura1*∆ pUDE737	This study
IME665	*MATa HIS3 LEU2 TRP1 URA3 can1*∆*::cas9-natNT2 ura1*∆ pUDE736	This study

### Molecular biology techniques

PCR was performed with Phusion High-Fidelity Polymerase (Thermo Fisher Scientific, Waltham MA) or DreamTaq (Thermo Fisher) for cloning and diagnostic purposes, respectively. Oligonucleotide primers (SI Table S1) were purchased from Sigma-Aldrich. PCR products amplified from plasmid templates were digested with FastDigest DpnI (Thermo Fisher) to avoid contamination with template DNA. Fragment sizes were analysed by electrophoresis on 1% (w/v) agarose gels. PCR products were purified with the GeneElute PCR Clean-Up Kit (Sigma-Aldrich) or the Zymoclean Gel DNA Recovery Kit (Zymo Research, Irvine CA). Plasmids were purified using with a GeneElute Plasmid Miniprep Kit (Sigma-Aldrich).

### Plasmid construction

Coding regions of DHO genes from *A. alternata* (A0A177DSV5) and *Sch. commune* (D8QKJ0), referred to as Aa*dho* and Sc*dho*, respectively, were derived from the UniProt Database (The UniProt Consortium 2019). Plasmids pUD709 (Aa*dho*) and pUD708 (Sc*dho*), carrying versions of these sequences that were codon optimised for expression in *S. cerevisiae* using the online GeneOptimizer tool (Raab et al. 2010) and obtained from GeneArt (Regensburg, Germany; Table 5). Coding regions were PCR amplified from these plasmids using primer pairs 12,365/12366 and 12363/12364, respectively. A pUDE63 backbone, containing a *TDH3* promoter and an *AHD1* terminator, was PCR amplified with primer pair 7823/7998, followed by Gibson Assembly (New England Biolabs, Ipswich MA; Gibson et al. 2009) with the Aa*dho* and Sc*dho* coding regions. The resulting plasmids pUDE737 (*TDH3*p-Aa*dho*-*ADH1*t) and pUDE736 (*TDH3*p-Sc*dho*-*ADH1*t) carried yeast expression cassettes for the two putative *dho* genes. pUDR348, which carries an expression cassette for a guide RNA targeting the *URA1* locus of *S. cerevisiae* IMX585 (*can1*∆::*cas9*-*natNT2*) was constructed by Gibson assembly of a pMEL13 backbone and 2 µm fragment as described previously (Mans et al. 2015). The pMEL13 backbone was amplified with PCR primer 6005 and the 2 µm fragment with primer pair 11,334/11335 (SI Table S2). *E. coli* XLI-Blue was transformed with the constructed plasmids and incubated for 5 min on ice, followed by 1 h incubation at 37 °C prior to plating on LB with ampicillin.

**Table 5:** Plasmids used in this study

Plasmid	Relevant characteristics	Purpose	Reference
pUD63	2 µm *ampR URA3 TDH3*p-*ADH1*t	Empty vector	de Kok et al. (2011)
pUD708	*ampR* Sc*dho*	Template	GeneArt
pUD709	*ampR* Aa*dho*	Template	GeneArt
pMEL13	2 µm *ampR kanMX* gRNA-*CAN1*	Template	Mans et al. (2015)
pUDE63	2 µm *ampR URA3 TDH3*p-*pgmB*-*ADH1*t	Template	de Kok et al. (2011)
pUDE736	2 µm *ampR URA3 TDH3*p-Sc*dho*-*ADH1*t	Expression Sc*dho*	This study
pUDE737	2 µm *ampR URA3 TDH3*p-Aa*dho*-*ADH1*t	Expression Aa*dho*	This study
pUDR348	2 µm *ampR kanMX* gRNA-*URA1*	Targeting *URA1*	This study

### Strain construction

*S. cerevisiae* strains were transformed with 1–2 µg plasmid DNA by the LiAc/SS-DNA/PEG-method (Gietz and Woods 2002). Strains IME410, IME414 and IME426 were constructed by transformation of CEN.PK 113-5D (ura3-52) with plasmids pUDE737 (Aa*dho*), pUDE736 (Sc*dho*) and pUD63 (empty vector), respectively, followed by selection on SMD plates and diagnostic PCR. Similarly, IME664 (*ura1*Δ mcAa*dho*) and IME665 (*ura1*Δ mcSc*dho*) were constructed by transforming *S. cerevisiae* IMK824 (*ura1*Δ) with pUDE737 (Aa*dho*) and pUDE736 (Sc*dho*), respectively. Cas9-mediated deletion or integration of genes in the Cas9-expressing *S. cerevisiae* strain IMX585 (*can1*∆*::cas9-natNT2*; Mans et al. 2015) was done according to Dicarlo et al. (2013). The *URA1* locus was targeted using the guide-RNA plasmid pUDR348. For construction of the *ura1* deletion strain IMK824, strain IMX585 was co-transformed with a repair oligonucleotide obtained by annealing oligonucleotides 11,336 and 11,337 (SI Table S1) and pUDR348. For integration of expression cassettes of the putative *dho* genes from *A. alternata* and *Sch. commune*, repair fragments containing these genes with upstream and downstream flanking *URA1* sequences, were amplified from pUDE736 and pUDE737, respectively, with primers 12,479 and 12,480. Integration of these fragments at the *URA1* locus yielded strains IMI433 (*ura1*∆*::*Aa*dho*) and IMI434 (*ura1*∆*::*Sc*dho*), respectively.

### Phylogeny of putative LkUra1 orthologs

For a systematic search for Ura1 orthologs, fungal proteomes (taxid 4751) available from the UniProt reference release 2019_02 (The UniProt Consortium 2019) were supplemented with sequences available from the UniProt trembl division for the following organisms: *Dekkera bruxellensis* (taxid 5007), *Komagataella phaffii* (taxid 981,350), *Komagataella pseudopastoris* (taxid 169,507), *Komagataella pastoris* (taxid 4922), *Ogataea polymorpha* (taxid 460,523), *Pichia membranifaciens* (taxid 763,406), *Pichia kudriavzevii* (taxid 4909), *Neocallimastix californiae* (taxid 1,754,190)*, Piromyces finnis* (taxid 1,754,191)*, Anaeromyces_robustus* (taxid 1,754,192) and *Piromyces* sp. E2 (taxid 73,868). The Ura1 sequence of *Lachancea kluyveri* CBS3082 (LkUra1, Q7Z892; Gojković et al. 2004) was used as query for a HMMER search of these sequences (Mistry et al. 2013). Cutoff values of 1e-5 were used, and hits were required to correspond to at least 75% of the query sequence length. From the resulting sequence, Ura1 orthologs were identified by calculating all possible co-ortholog sets with proteinortho v6.0.25 (Lechner et al. 2011) running diamond v2.0.8 (Buchfink et al. 2021). The resulting 203 fungal Ura1 orthologs (Dataset_S01) were subjected to a multiple sequence alignment using MAFFT v7.40286 (Katoh and Standley 2013) in “einsi” mode. Alignments were trimmed using trimAl v1.287 (Capella-Gutiérrez et al. 2009) in “gappyout” mode, and used to build a phylogenetic tree with RAxML-NG v0.8.188 (Kozlov et al. 2019) using 10 random and 10 parsimony starting trees, 100 Felsestein Bootstrap replicates, and LG model. The resulting phylogenetic tree (Dataset_S02) was visualised using iTOL (Interactive Tree Of Life) tool v6 (Letunic and Bork 2016). To search for fungal homologs of dihydropyrimidinase (DHP), a protein search (blastp; Camacho et al. 2009) was performed on fungal proteomes (taxid: 4751) with the DHP sequence of *Lachancea kluyveri* (Uniprot KB accession: Q9P903) as query. Resulting protein sequences with an E-value below 1e-5 are provided in Dataset_S03.

### Aerobic growth experiments

Shake-flask cultures were grown in 500-mL flasks containing 100 mL of medium. A frozen stock culture was used to inoculate a pre-culture on SMD + ura. Upon reaching stationary phase, a 1-mL sample of this pre-culture was transferred to a second pre-culture on SMD + ura0.1. This second pre-culture was grown until late exponential phase, centrifuged (5 min at 3000 g), washed twice with sterile demineralised water and used as inoculum for either plate or shake-flask growth experiments. Shake flasks were incubated in an Innova incubator (New Brunswick Scientific), operated at 30 °C and at 200 rpm. Agar plates were incubated for 2 d at 30 °C. For cell sorting experiments, SMD was used for the first pre-culture, while the second culture was grown on either 1 mL SMD + ura0.1 supplemented with 5F-dhu or on 1 mL SMD + ura in a sterile 1.5-mL Eppendorf tube (Eppendorf Corporate, Hamburg, Germany).

### Anaerobic cultivation

Anaerobic cultures were grown in a Bactron 300–2 anaerobic workstation (Sheldon Manufacturing Inc, Cornelius OR) equipped with a Pd-catalyst and filled with a mixed gas atmosphere (85% N_2_, 10% CO_2_, 5% H_2_). To minimise oxygen entry, the protocols described by Mooiman et al. (2021) for cultivation in anaerobic chambers were followed. Flasks were placed on an IKA KS 260 basic orbital shaker (IKA-Werke, Staufen im Breisgau, Germany) at 240 rpm and temperature in the workspace was maintained at 30 °C. Strains were pre-grown aerobically, centrifuged (3000 g, 5 min), washed with sterile demineralised water and used to inoculate medium in the anaerobic workspace. The initial optical density at 600 nm of anaerobic pre-cultures, which were grown on SMUD + dhu0.1 or SMUD + ura0.1 was 0.2. Stationary-phase pre-cultures were used to start subsequent anaerobic growth experiments on SMUD or SMUD + dhu. All anaerobic (pre-)cultures were supplemented with Tween 80 and ergosterol.

### Analytical methods and calculation

Optical density of aerobic cultures was measured at 660 nm (OD_660_) with a Jenway 7200 Spectrophotometer (Bibby Scientific, Staffordshire, UK). Maximum specific growth rates were calculated from the slope of ln OD_660_ versus time during the exponential phase, considering at least six time points. Optical density measurements in anaerobic cultures were performed at 600 nm with an Ultrospec 10 cell density meter (Biochrom, Harvard Biosience, Holliston MA) placed in the anaerobic workstation. For spot-plate experiments, cell counts in late-exponential-phase shake-flask cultures were first determined using a Z2 Coulter Counter Analyzer (Beckman Coulter Life Sciences, Indianapolis IN) following the manufacturer’s protocol. Prior to analysis, cultures were diluted one 100-fold with Isoton II (Beckman Coulter, Brea CA), followed by five replicate cell counts on each sample.

#### Enzyme assays

Cell extracts were prepared by sonication and centrifugation (Vuralhan et al. 2005) of cells grown aerobically on SMUD + dhu until late-exponential phase. Protein concentrations in cell extracts were determined with the Lowry method (Lowry et al. 1951) and with bovine serum albumin as reference. Oxygen consumption was measured with a Clark-type oxygen electrode at 30 °C in 0.1 M potassium phosphate buffer (pH 7.5) with 2 mM MgCl_2_ as described previously (Visser et al. 1995), using two independently prepared cell extracts for each yeast strain. Air-saturated buffer (236 μM O_2_) was used to calibrate the oxygen electrode. Assays were performed in a reaction volume of 4 mL and started by addition of dihydrothymine or dihydrouracil (final concentration 1 mM). Production of H_2_O_2_ was tested by addition of 4 μL catalase suspension from bovine liver (500 U mL^−1^; Sigma Aldrich) during the reaction.

#### Viability and plasmid loss assays

Single cells were sorted with a BD FACSAria II SORP cell sorter (BD Biosciences) as described previously (Gorter de Vries et al. 2019), with the modification that cells were sorted based on forward scatter (FSC) and side scatter (SSC) instead of fluorescence. Late-exponential-phase cultures grown on either SMD + ura + 5F-dhu or on SMD were diluted ten-fold in sterile Isoton II (Beckman Coulter) and > 10^5^ events were analysed. The FSC of analysed events was plotted against the SSC to set the sorting region such that bias for cell morphology was avoided. Unless indicated differently, 4 × 384 single cells were sorted from each culture using the single-cell sorting mask (0/32/16), and placed on two SMD and two SMD + ura plates.

## Supplementary Information

Below is the link to the electronic supplementary material.

Supplementary file1 (DOCX 386 KB)

## Data Availability

All data generated or analysed in this study are included in the article and supplementary information files.
