# Supplementary material for: Identification of fungal dihydrouracil-oxidase genes by expression in Saccharomyces cerevisiae
Source: Antonie Van Leeuwenhoek. 2022 Oct 14;115(11):1363–78. doi: 10.1007/s10482-022-01779-9 (PMC9585004; doi:10.1007/s10482-022-01779-9)
Supplement: Supplementary file 1 — Supplementary file1 (DOCX 386 KB) [file 10482_2022_1779_MOESM1_ESM.docx]

# Supplementary Information

# Identification of fungal dihydrouracil-oxidase genes by expression in *Saccharomyces cerevisiae*

Jonna Bouwknegt^1^, Aurin M. Vos^1^, Raúl A. Ortiz Merino, Daphne C. van Cuylenburg-Oude Vrielink, Marijke A. H. Luttik and Jack T. Pronk*

Department of Biotechnology, Delft University of Technology, van der Maasweg 9, 2629 HZ Delft, the Netherlands

Manuscript for publication in Antonie van Leeuwenhoek

Category: Fungal and Yeast Microbiology

*Correspondence: Jack T. Pronk, e-mail [j.t.pronk@tudelft.nl](mailto:j.t.pronk@tudelft.nl), tel +31 15 2782416

**This PDF file contains:**

Figure S1

Table S1-S2

**Separate datasets:**

Dataset S01: fasta file multiple sequence alignment

Dataset S02: raw phylogenetic tree data

Dataset S03: fasta file blast search


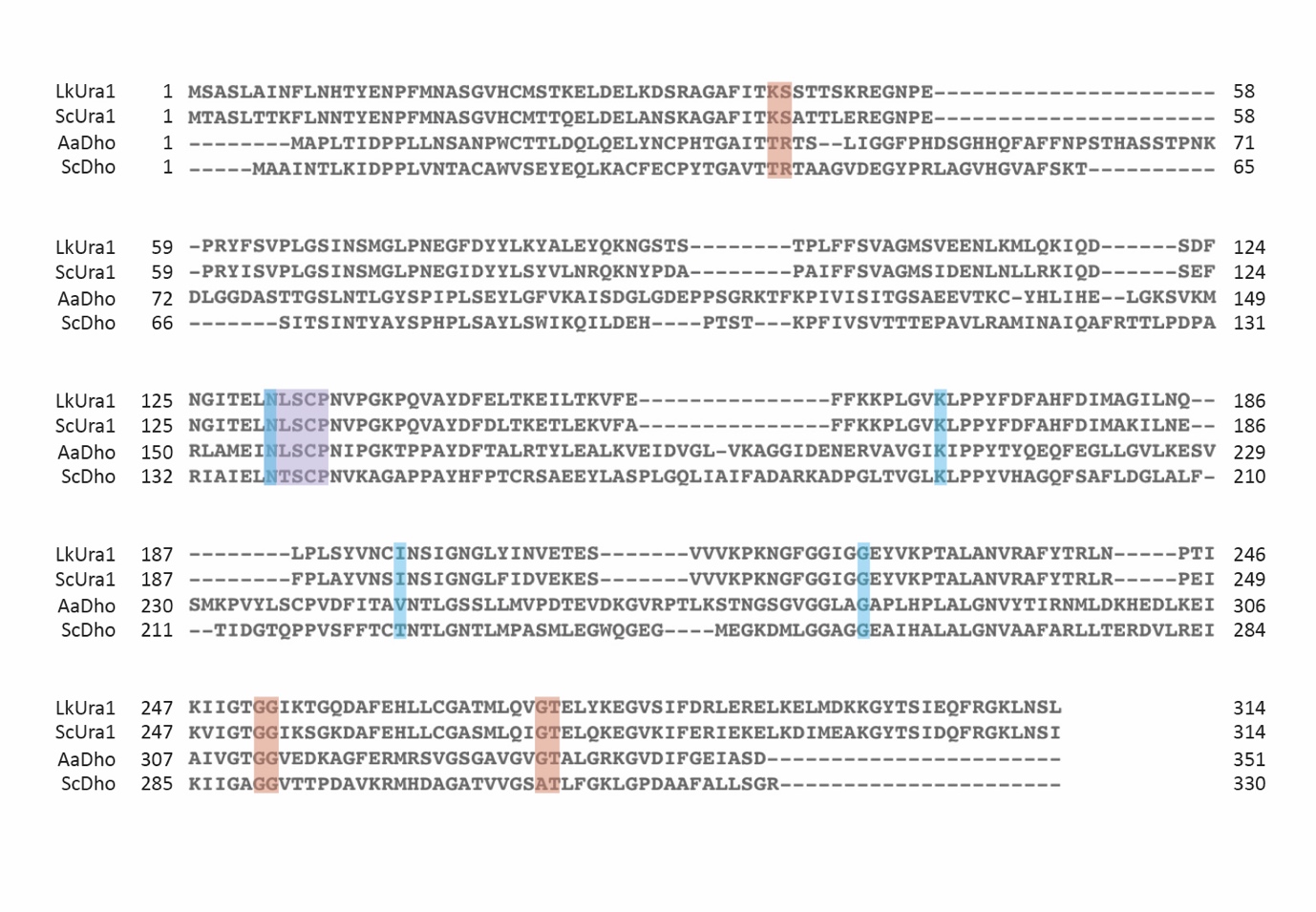
**Figure S1. Multiple sequence alignment of Ura1 enzymes and DHO enzymes.** Class-I-A protein sequences of *S. cerevisiae* (ScUra1) and *L. kluyveri* (LkUra1) and DHO proteins of *A. alternata* (AaDho) and *Sch. commune* (ScDho) were subjected to a multiple sequence alignment in Clustal Omega using default settings. Based on protein sequence similarity, predicted functional sites (The UniProt Consortium 2019) were shown. Purple; active site (NxSCP), Blue; binding sites FMN, Red; nucleotide binding of FMN.

**Table S1. 10 highest scoring bacterial homologs for a blastp search with Ura1-orthologs from mucormycota a.** A blastp (NCBI; (Camacho et al. 2009)) search in available proteomes of bacteria (taxid:2) was performed using Ura1-orthologs of Mucoromycota fungi (Figure 1), *S. cerevisiae* (ScUra1) or *L. kluyveri* (LkUra1) as query. The ten proteins with highest similarity and their respective host, were presented in this table. Results are presented with the host organism (top), GenBank accession number of the protein (middle), and E-value (bottom).

| Query  *Organism*  Protein | *Rhizopus azygosporus*  A0A367JNI9 | *Rhizopus azygosporus*  A0A367JHE7 | *Rhizopus microsporus*  A0A2G4SIK6 | *Rhizopus delamar*  I1BT22 | *Parasitella parasiticas*  A0A0B7NIC1 | *Mucor ambiguus*  A0A0C9MDX3 | *Absidia glauca*  A0A168QAJ3 | *Phycomyces blakeleeanus*  A0A167N250 | *Saccharomyces cerevisiae*  P28272 | *Lachancea kluyveri*  Q7Z892 |
| --- | --- | --- | --- | --- | --- | --- | --- | --- | --- | --- |
| 1 | *Laribacter honkongensis*  [WP_027824372.1](https://www.ncbi.nlm.nih.gov/protein/WP_027824372.1?report=genbank&log$=prottop&blast_rank=1&RID=G26JVUGB013)  4·10^-137^ | *Laribacter honkongensis*  [WP_027824372.1](https://www.ncbi.nlm.nih.gov/protein/WP_027824372.1?report=genbank&log$=prottop&blast_rank=1&RID=G26JVUGB013)  4·10^-137^ | *Crenobacter cavernae*  [WP_129210658.1](https://www.ncbi.nlm.nih.gov/protein/WP_129210658.1?report=genbank&log$=prottop&blast_rank=1&RID=G28AFFD201R)  4·10^-136^ | *Crenobacter cavernae*  [WP_129210658.1](https://www.ncbi.nlm.nih.gov/protein/WP_129210658.1?report=genbank&log$=prottop&blast_rank=3&RID=G26JVUGB013)  5·10^-137^ | *Crenobacter cavernae*  [WP_115431880.1](https://www.ncbi.nlm.nih.gov/protein/WP_115431880.1?report=genbank&log$=prottop&blast_rank=4&RID=G26JVUGB013)  5·10^-137^ | *Crenobacter cavernae*  [WP_129210658.1](https://www.ncbi.nlm.nih.gov/protein/WP_129210658.1?report=genbank&log$=prottop&blast_rank=3&RID=G26JVUGB013)  5·10^-138^ | *Jeongeupia naejangsanensis*  [WP_203537192.1](https://www.ncbi.nlm.nih.gov/protein/WP_203537192.1?report=genbank&log$=prottop&blast_rank=5&RID=G28AFFD201R)  5·10^-138^ | *Laribacter* sp.  [MBP8813637.1](https://www.ncbi.nlm.nih.gov/protein/MBP8813637.1?report=genbank&log$=prottop&blast_rank=5&RID=G5GVS4GS013)  2·10^-135^ | *Enterococcus* sp. CU12B  [KAF1305604.1](https://www.ncbi.nlm.nih.gov/protein/KAF1305604.1?report=genbank&log$=prottop&blast_rank=1&RID=G5VGP9A6016)  6·10^-173^ | *Enterococcus mediterraneensis*  [WP_122646993.1](https://www.ncbi.nlm.nih.gov/protein/WP_122646993.1?report=genbank&log$=prottop&blast_rank=1&RID=G5WE0FGS016)  2·10^-178^ |
| 2 | *Laribacter honkongensis*  [WP_012696339.1](https://www.ncbi.nlm.nih.gov/protein/WP_012696339.1?report=genbank&log$=prottop&blast_rank=2&RID=G26JVUGB013)  7·10^-137^ | *Laribacter honkongensis*  [WP_012696339.1](https://www.ncbi.nlm.nih.gov/protein/WP_012696339.1?report=genbank&log$=prottop&blast_rank=2&RID=G26JVUGB013)  7·10^-137^ | *Laribacter honkongensis*  [WP_027824372.1](https://www.ncbi.nlm.nih.gov/protein/WP_027824372.1?report=genbank&log$=prottop&blast_rank=1&RID=G26JVUGB013)  5·10^-136^ | *Crenobacter cavernae*  [WP_115431880.1](https://www.ncbi.nlm.nih.gov/protein/WP_115431880.1?report=genbank&log$=prottop&blast_rank=4&RID=G26JVUGB013)  2·10^-136^ | *Crenobacter cavernae*  [WP_129210658.1](https://www.ncbi.nlm.nih.gov/protein/WP_129210658.1?report=genbank&log$=prottop&blast_rank=3&RID=G26JVUGB013)  2·10^-136^ | *Crenobacter cavernae*  [WP_115431880.1](https://www.ncbi.nlm.nih.gov/protein/WP_115431880.1?report=genbank&log$=prottop&blast_rank=4&RID=G26JVUGB013)  2·10^-137^ | *Laribacter* sp.  [MBP8813637.1](https://www.ncbi.nlm.nih.gov/protein/MBP8813637.1?report=genbank&log$=prottop&blast_rank=5&RID=G5GVS4GS013)  2·10^-137^ | *Laribacter honkongensis*  [WP_027824372.1](https://www.ncbi.nlm.nih.gov/protein/WP_027824372.1?report=genbank&log$=prottop&blast_rank=1&RID=G26JVUGB013)  7·10^-135^ | *Enterococcus* sp. CU12B  [WP_202622260.1](https://www.ncbi.nlm.nih.gov/protein/WP_202622260.1?report=genbank&log$=prottop&blast_rank=2&RID=G5VGP9A6016)  7·10^-173^ | *Enterococcus massiliensis*  [WP_048602930.1](https://www.ncbi.nlm.nih.gov/protein/WP_048602930.1?report=genbank&log$=prottop&blast_rank=2&RID=G5WE0FGS016)  3·10^-176^ |
| 3 | *Crenobacter cavernae*  [WP_129210658.1](https://www.ncbi.nlm.nih.gov/protein/WP_129210658.1?report=genbank&log$=prottop&blast_rank=3&RID=G26JVUGB013)  1·10^-136^ | *Crenobacter cavernae*  [WP_129210658.1](https://www.ncbi.nlm.nih.gov/protein/WP_129210658.1?report=genbank&log$=prottop&blast_rank=3&RID=G26JVUGB013)  4·10^-136^ | *Laribacter honkongensis*  [WP_012696339.1](https://www.ncbi.nlm.nih.gov/protein/WP_012696339.1?report=genbank&log$=prottop&blast_rank=2&RID=G26JVUGB013)  1·10^-135^ | *Jeongeupia* sp. USM3  [WP_070525525.1](https://www.ncbi.nlm.nih.gov/protein/WP_070525525.1?report=genbank&log$=prottop&blast_rank=3&RID=G28WXZDM016)  5·10^-136^ | *Jeongeupia naejangsanensis*  [WP_203537192.1](https://www.ncbi.nlm.nih.gov/protein/WP_203537192.1?report=genbank&log$=prottop&blast_rank=5&RID=G28AFFD201R)  6·10^-136^ | *Laribacter honkongensis*  [WP_027824372.1](https://www.ncbi.nlm.nih.gov/protein/WP_027824372.1?report=genbank&log$=prottop&blast_rank=1&RID=G26JVUGB013)  3·10^-136^ | *Chitiniphilus shinanonensis*  [WP_040430830.1](https://www.ncbi.nlm.nih.gov/protein/WP_040430830.1?report=genbank&log$=prottop&blast_rank=3&RID=G2A5VDU4016)  3·10^-137^ | *Laribacter honkongensis*  [WP_012696339.1](https://www.ncbi.nlm.nih.gov/protein/WP_012696339.1?report=genbank&log$=prottop&blast_rank=2&RID=G26JVUGB013)  2·10^-134^ | *Enterococcus faecalis*  [WP_034862897.1](https://www.ncbi.nlm.nih.gov/protein/WP_034862897.1?report=genbank&log$=prottop&blast_rank=3&RID=G5VGP9A6016)  2·10^-172^ | *Enterococcus* Sp. DIV2402  [WP_207940501.1](https://www.ncbi.nlm.nih.gov/protein/WP_207940501.1?report=genbank&log$=prottop&blast_rank=3&RID=G5WE0FGS016)  1·10^-174^ |
| 4 | *Crenobacter cavernae*  [WP_115431880.1](https://www.ncbi.nlm.nih.gov/protein/WP_115431880.1?report=genbank&log$=prottop&blast_rank=4&RID=G26JVUGB013)  2·10^-135^ | *Crenobacter cavernae*  [WP_115431880.1](https://www.ncbi.nlm.nih.gov/protein/WP_115431880.1?report=genbank&log$=prottop&blast_rank=4&RID=G26JVUGB013)  1·10^-135^ | *Crenobacter cavernae*  [WP_115431880.1](https://www.ncbi.nlm.nih.gov/protein/WP_115431880.1?report=genbank&log$=prottop&blast_rank=4&RID=G26JVUGB013)  2·10^-135^ | *Jeongeupia naejangsanensis*  [WP_203537192.1](https://www.ncbi.nlm.nih.gov/protein/WP_203537192.1?report=genbank&log$=prottop&blast_rank=5&RID=G28AFFD201R)  5·10^-136^ | *Laribacter honkongensis*  [WP_027824372.1](https://www.ncbi.nlm.nih.gov/protein/WP_027824372.1?report=genbank&log$=prottop&blast_rank=1&RID=G26JVUGB013)  6·10^-135^ | *Laribacter honkongensis*  [WP_012696339.1](https://www.ncbi.nlm.nih.gov/protein/WP_012696339.1?report=genbank&log$=prottop&blast_rank=2&RID=G26JVUGB013)  5·10^-136^ | *Jeongeupia* sp. USM3  [WP_070525525.1](https://www.ncbi.nlm.nih.gov/protein/WP_070525525.1?report=genbank&log$=prottop&blast_rank=3&RID=G28WXZDM016)  3·10^-136^ | *Jeongeupia naejangsanensis*  [WP_203537192.1](https://www.ncbi.nlm.nih.gov/protein/WP_203537192.1?report=genbank&log$=prottop&blast_rank=5&RID=G28AFFD201R)  4·10^-130^ | *Enterococcus gallinarum* [WP_117474532.1](https://www.ncbi.nlm.nih.gov/protein/WP_117474532.1?report=genbank&log$=prottop&blast_rank=4&RID=G5VGP9A6016)  3·10^-172^ | Unclassified *Enterococcus*  [WP_165005935.1](https://www.ncbi.nlm.nih.gov/protein/WP_165005935.1?report=genbank&log$=prottop&blast_rank=4&RID=G5WE0FGS016)  2·10^-174^ |
| 5 | *Laribacter* sp.  [MBP8813637.1](https://www.ncbi.nlm.nih.gov/protein/MBP8813637.1?report=genbank&log$=prottop&blast_rank=5&RID=G5GVS4GS013)  3·10^-135^ | *Laribacter* sp.  [MBP8813637.1](https://www.ncbi.nlm.nih.gov/protein/MBP8813637.1?report=genbank&log$=prottop&blast_rank=5&RID=G5GVS4GS013)  2·10^-135^ | *Jeongeupia naejangsanensis*  [WP_203537192.1](https://www.ncbi.nlm.nih.gov/protein/WP_203537192.1?report=genbank&log$=prottop&blast_rank=5&RID=G28AFFD201R)  2·10^-134^ | *Jeongeupia chitinilytica*  WP_189458989.1  2·10^-134^ | *Jeongeupia* sp. USM3  [WP_070525525.1](https://www.ncbi.nlm.nih.gov/protein/WP_070525525.1?report=genbank&log$=prottop&blast_rank=3&RID=G28WXZDM016)  6·10^-135^ | *Crenobacter luteus*  [WP_082824196.1](https://www.ncbi.nlm.nih.gov/protein/WP_082824196.1?report=genbank&log$=prottop&blast_rank=9&RID=G5GVS4GS013)  8·10^-136^ | *Laribacter honkongensis*  [WP_012696339.1](https://www.ncbi.nlm.nih.gov/protein/WP_012696339.1?report=genbank&log$=prottop&blast_rank=2&RID=G26JVUGB013)  4·10^-136^ | *Jeongeupia* sp. USM3  [WP_070525525.1](https://www.ncbi.nlm.nih.gov/protein/WP_070525525.1?report=genbank&log$=prottop&blast_rank=3&RID=G28WXZDM016)  1·10^-129^ | *Enterococcus*  [WP_029486858.1](https://www.ncbi.nlm.nih.gov/protein/WP_029486858.1?report=genbank&log$=prottop&blast_rank=5&RID=G5VGP9A6016)  3·10^-172^ | *Enterococcus* sp. DIV1271a  [WP_207699663.1](https://www.ncbi.nlm.nih.gov/protein/WP_207699663.1?report=genbank&log$=prottop&blast_rank=5&RID=G5WE0FGS016)  2·10^-173^ |
| 6 | *Jeongeupia chitinilytica*  [WP_189458989.1](https://www.ncbi.nlm.nih.gov/protein/WP_189458989.1?report=genbank&log$=prottop&blast_rank=8&RID=G5GVS4GS013)  1·10^-134^ | *Jeongeupia naejangsanensis*  [WP_203537192.1](https://www.ncbi.nlm.nih.gov/protein/WP_203537192.1?report=genbank&log$=prottop&blast_rank=5&RID=G28AFFD201R)  1·10^-134^ | *Laribacter* sp.  [MBP8813637.1](https://www.ncbi.nlm.nih.gov/protein/MBP8813637.1?report=genbank&log$=prottop&blast_rank=5&RID=G5GVS4GS013)  3·10^-134^ | *Laribacter honkongensis*  [WP_027824372.1](https://www.ncbi.nlm.nih.gov/protein/WP_027824372.1?report=genbank&log$=prottop&blast_rank=1&RID=G26JVUGB013)  5·10^-134^ | *Crenobacter luteus*  [WP_082824196.1](https://www.ncbi.nlm.nih.gov/protein/WP_082824196.1?report=genbank&log$=prottop&blast_rank=9&RID=G5GVS4GS013)  9·10^-135^ | *Crenobacter luteus*  [WP_131862996.1](https://www.ncbi.nlm.nih.gov/protein/WP_131862996.1?report=genbank&log$=prottop&blast_rank=10&RID=G5GVS4GS013)  1·10^-135^ | *Crenobacter cavernae*  [WP_115431880.1](https://www.ncbi.nlm.nih.gov/protein/WP_115431880.1?report=genbank&log$=prottop&blast_rank=4&RID=G26JVUGB013)  5·10^-136^ | *Crenobacter cavernae*  [WP_115431880.1](https://www.ncbi.nlm.nih.gov/protein/WP_115431880.1?report=genbank&log$=prottop&blast_rank=4&RID=G26JVUGB013)  7·10^-129^ | *Enterococcus* sp. DIV2402  [WP_207940501.1](https://www.ncbi.nlm.nih.gov/protein/WP_207940501.1?report=genbank&log$=prottop&blast_rank=6&RID=G5VGP9A6016)  1·10^-171^ | *Enterococcus* sp. DIV1298c  [WP_207115710.1](https://www.ncbi.nlm.nih.gov/protein/WP_207115710.1?report=genbank&log$=prottop&blast_rank=6&RID=G5WE0FGS016)  3·10^-173^ |
| 7 | *Crenobacter luteus*  [WP_082824196.1](https://www.ncbi.nlm.nih.gov/protein/WP_082824196.1?report=genbank&log$=prottop&blast_rank=9&RID=G5GVS4GS013)  9·10^-134^ | *Jeongeupia chitinilytica*  [WP_189458989.1](https://www.ncbi.nlm.nih.gov/protein/WP_189458989.1?report=genbank&log$=prottop&blast_rank=8&RID=G5GVS4GS013)  4·10^-134^ | *Jeongeupia* sp. USM3  [WP_070525525.1](https://www.ncbi.nlm.nih.gov/protein/WP_070525525.1?report=genbank&log$=prottop&blast_rank=3&RID=G28WXZDM016)  1·10^-133^ | *Laribacter honkongensis*  [WP_012696339.1](https://www.ncbi.nlm.nih.gov/protein/WP_012696339.1?report=genbank&log$=prottop&blast_rank=2&RID=G26JVUGB013)  9·10^-134^ | *Laribacter honkongensis*  [WP_012696339.1](https://www.ncbi.nlm.nih.gov/protein/WP_012696339.1?report=genbank&log$=prottop&blast_rank=2&RID=G26JVUGB013)  1·10^-134^ | *Laribacter* sp.  [MBP8813637.1](https://www.ncbi.nlm.nih.gov/protein/MBP8813637.1?report=genbank&log$=prottop&blast_rank=5&RID=G5GVS4GS013)  2·10^-135^ | *Crenobacter cavernae*  [WP_129210658.1](https://www.ncbi.nlm.nih.gov/protein/WP_129210658.1?report=genbank&log$=prottop&blast_rank=3&RID=G26JVUGB013)  1·10^-135^ | *Crenobacter cavernae*  [WP_129210658.1](https://www.ncbi.nlm.nih.gov/protein/WP_129210658.1?report=genbank&log$=prottop&blast_rank=3&RID=G26JVUGB013)  2·10^-128^ | *Enterococcus*  [WP_003127297.1](https://www.ncbi.nlm.nih.gov/protein/WP_003127297.1?report=genbank&log$=prottop&blast_rank=7&RID=G5VGP9A6016)  2·10^-171^ | *Enterococcus*  [WP_071867076.1](https://www.ncbi.nlm.nih.gov/protein/WP_071867076.1?report=genbank&log$=prottop&blast_rank=7&RID=G5WE0FGS016)  3·10^-173^ |
| 8 | *Crenobacter luteus*  [WP_131862996.1](https://www.ncbi.nlm.nih.gov/protein/WP_131862996.1?report=genbank&log$=prottop&blast_rank=10&RID=G5GVS4GS013)  6·10^-132^ | *Crenobacter luteus*  [WP_082824196.1](https://www.ncbi.nlm.nih.gov/protein/WP_082824196.1?report=genbank&log$=prottop&blast_rank=9&RID=G5GVS4GS013)  1·10^-132^ | *Crenobacter luteus*  [WP_082824196.1](https://www.ncbi.nlm.nih.gov/protein/WP_082824196.1?report=genbank&log$=prottop&blast_rank=9&RID=G5GVS4GS013)  4·10^-132^ | *Laribacter* sp.  [MBP8813637.1](https://www.ncbi.nlm.nih.gov/protein/MBP8813637.1?report=genbank&log$=prottop&blast_rank=5&RID=G5GVS4GS013)  4·10^-133^ | *Crenobacter luteus*  [WP_131862996.1](https://www.ncbi.nlm.nih.gov/protein/WP_131862996.1?report=genbank&log$=prottop&blast_rank=10&RID=G5GVS4GS013)  1·10^-134^ | *Jeongeupia naejangsanensis*  [WP_203537192.1](https://www.ncbi.nlm.nih.gov/protein/WP_203537192.1?report=genbank&log$=prottop&blast_rank=5&RID=G28AFFD201R)  3·10^-134^ | *Laribacter honkongensis*  [WP_027824372.1](https://www.ncbi.nlm.nih.gov/protein/WP_027824372.1?report=genbank&log$=prottop&blast_rank=1&RID=G26JVUGB013)  2·10^-135^ | *Chitinibacter tainanensis*  [WP_034619876.1](https://www.ncbi.nlm.nih.gov/protein/WP_034619876.1?report=genbank&log$=prottop&blast_rank=8&RID=G5N5R2AT013)  3·10^-128^ | *Enterococcus*  [WP_138371833.1](https://www.ncbi.nlm.nih.gov/protein/WP_138371833.1?report=genbank&log$=prottop&blast_rank=8&RID=G5VGP9A6016)  3·10^-171^ | *Enterococcus* DIV1094  [WP_206854181.1](https://www.ncbi.nlm.nih.gov/protein/WP_206854181.1?report=genbank&log$=prottop&blast_rank=8&RID=G5WE0FGS016)  4·10^-173^ |
| 9 | *Jeongeupia* sp. HS-3  [WP_200917361.1](https://www.ncbi.nlm.nih.gov/protein/WP_200917361.1?report=genbank&log$=prottop&blast_rank=11&RID=G5GVS4GS013)  1·10^-131^ | *Crenobacter luteus*  [WP_131862996.1](https://www.ncbi.nlm.nih.gov/protein/WP_131862996.1?report=genbank&log$=prottop&blast_rank=10&RID=G5GVS4GS013)  7·10^-132^ | *Crenobacter luteus*  [WP_131862996.1](https://www.ncbi.nlm.nih.gov/protein/WP_131862996.1?report=genbank&log$=prottop&blast_rank=10&RID=G5GVS4GS013)  1·10^-131^ | *Crenobacter luteus*  [WP_082824196.1](https://www.ncbi.nlm.nih.gov/protein/WP_082824196.1?report=genbank&log$=prottop&blast_rank=9&RID=G5GVS4GS013)  1·10^-131^ | *Jeongeupia chitinilytica*  [WP_189458989.1](https://www.ncbi.nlm.nih.gov/protein/WP_189458989.1?report=genbank&log$=prottop&blast_rank=8&RID=G5GVS4GS013)  2·10^-134^ | *Jeongeupia* sp. USM3  [WP_070525525.1](https://www.ncbi.nlm.nih.gov/protein/WP_070525525.1?report=genbank&log$=prottop&blast_rank=3&RID=G28WXZDM016)  4·10^-134^ | *Chitiniphilus eburneus*  [WP_136774475.1](https://www.ncbi.nlm.nih.gov/protein/WP_136774475.1?report=genbank&log$=prottop&blast_rank=9&RID=G5MPTRMA016)  2·10^-135^ | *Chitinibacter* sp. 2T18  [WP_179358028.1](https://www.ncbi.nlm.nih.gov/protein/WP_179358028.1?report=genbank&log$=prottop&blast_rank=10&RID=G5KK80C701R)  4·10^-128^ | *Enterococcus mundtii*  [WP_195339280.1](https://www.ncbi.nlm.nih.gov/protein/WP_195339280.1?report=genbank&log$=prottop&blast_rank=9&RID=G5VGP9A6016)  3·10^-171^ | *Enterococcus mundtii*  [WP_115357638.1](https://www.ncbi.nlm.nih.gov/protein/WP_115357638.1?report=genbank&log$=prottop&blast_rank=9&RID=G5WE0FGS016)  4·10^-173^ |
| 10 | *Uliginosibacterium* sp.  [MBK9218298.1](https://www.ncbi.nlm.nih.gov/protein/MBK9218298.1?report=genbank&log$=prottop&blast_rank=12&RID=G5GVS4GS013)  3·10^-131^ | *Uliginosibacterium* sp.  [MBK9218298.1](https://www.ncbi.nlm.nih.gov/protein/MBK9218298.1?report=genbank&log$=prottop&blast_rank=12&RID=G5GVS4GS013)  9·10^-132^ | *Uliginosibacterium* sp.  [MBK9218298.1](https://www.ncbi.nlm.nih.gov/protein/MBK9218298.1?report=genbank&log$=prottop&blast_rank=12&RID=G5GVS4GS013)  1·10^-131^ | *Crenobacter luteus*  [WP_131862996.1](https://www.ncbi.nlm.nih.gov/protein/WP_131862996.1?report=genbank&log$=prottop&blast_rank=10&RID=G5GVS4GS013)  1·10^-131^ | *Chitinibacter* sp. 2T18  [WP_179358028.1](https://www.ncbi.nlm.nih.gov/protein/WP_179358028.1?report=genbank&log$=prottop&blast_rank=10&RID=G5KK80C701R)  3·10^-133^ | *Crenobacter luteus*  [KZE30316.1](https://www.ncbi.nlm.nih.gov/protein/KZE30316.1?report=genbank&log$=prottop&blast_rank=10&RID=G5M1CWT6013)  4·10^-133^ | *Jeongeupia chitinilytica*  [WP_189458989.1](https://www.ncbi.nlm.nih.gov/protein/WP_189458989.1?report=genbank&log$=prottop&blast_rank=8&RID=G5GVS4GS013)  4·10^-135^ | *Uliginosibacterium* sp.  [MBK9218298.1](https://www.ncbi.nlm.nih.gov/protein/MBK9218298.1?report=genbank&log$=prottop&blast_rank=12&RID=G5GVS4GS013)  4·10^-128^ | *Enterococcus gallinarum* [WP_156247377.1](https://www.ncbi.nlm.nih.gov/protein/WP_156247377.1?report=genbank&log$=prottop&blast_rank=10&RID=G5VGP9A6016)  4·10^-171^ | *Enterococcus mundtii* [WP_074799017.1](https://www.ncbi.nlm.nih.gov/protein/WP_074799017.1?report=genbank&log$=prottop&blast_rank=10&RID=G5WE0FGS016)  4·10^-173^ |

**Table S2. Oligonucleotides used in this study.**

| **Number** | **5' sequence** | | | | | |
| --- | --- | --- | --- | --- | --- | --- |
| 6005 | GATCATTTATCTTTCACTGCGGAGAAG | |  |  |  |  |
| 7823 | ATCCGTCGAAACTAAGTTCTGG |  |  |  |  |  |
| 7998 | AAGCTTATCGATACCGTCGACC |  |  |  |  |  |
| 11334 | TGCGCATGTTTCGGCGTTCGAAACTTCTCCGCAGTGAAAGATAAATGATCGGTACAGAATTACAAAAAGAGTTTTAGAGCTAGAAATAGCAAGTTAAAATAAGGCTAGTCCGTTATCAAC | | | | | |
| 11335 | GTTGATAACGGACTAGCCTTATTTTAACTTGCTATTTCTAGCTCTAAAACTCTTTTTGTAATTCTGTACCGATCATTTATCTTTCACTGCGGAGAAGTTTCGAACGCCGAAACATGCGCA | | | | | |
| 11336 | TAGACGTTTCAAATAGATATACACAATTACTCAAAAAAAAATTGAACTTCCGTACCAAACTTATCGGAATTTGAAGAACTATGATGGGACAAGTTATGGAAGAGTGTTAAGATTCGTATG | | | | | |
| 11337 | CATACGAATCTTAACACTCTTCCATAACTTGTCCCATCATAGTTCTTCAAATTCCGATAAGTTTGGTACGGAAGTTCAATTTTTTTTTGAGTAATTGTGTATATCTATTTGAAACGTCTA | | | | | |
| 12363 | AGAACTTAGTTTCGACGGATGCCACGTGTCTTGTCCAGAG | | | |  |  |
| 12364 | TCGACGGTATCGATAAGCTTGTGCTCCAGGTACCTTATCTAC | | | |  |  |
| 12365 | AGAACTTAGTTTCGACGGATCAGAGCTCATGGCTCCATTGAC | | | |  |  |
| 12366 | TCGACGGTATCGATAAGCTTTCTTGTGCTCCAGGTACCTTAG | | | |  |  |
| 12479 | TAGACGTTTCAAATAGATATACACAATTACTCAAAAAAAAATTGAACTTCCGTACCAAACTACCCGATACCGGTGATGGC | | | | | |
| 12480 | CATACGAATCTTAACACTCTTCCATAACTTGTCCCATCATAGTTCTTCAAATTCCGATAAACCCTCACTAAAGGGAACAAAAGC | | | | | |
